# Biochemical characteristics of the chondrocyte-enriched SNORC protein and its transcriptional regulation by SOX9

**DOI:** 10.1038/s41598-020-64640-x

**Published:** 2020-05-08

**Authors:** Prashant Kumar Jaiswal, Latifa Aljebali, Marie-Hélène Gaumond, Chun-do Oh, Hideyo Yasuda, Pierre Moffatt

**Affiliations:** 1 Shriners Hospitals for Children – Canada, Montreal, Quebec, Canada; 2 Department of Human Genetics, McGill University, Montreal, Quebec, Canada; 3 Research Institute of the McGill University Health Centre, Montreal, Quebec, Canada; 4 Department of Orthopedic Surgery, Rush University Medical Center, Chicago, IL 60612 USA; 5 Department of Genetics, University of Texas, MD Anderson Cancer Center, Houston, TX 77030 USA

**Keywords:** Biochemistry, Molecular biology

## Abstract

*Snorc* (Small NOvel Rich in Cartilage) has been identified as a chondrocyte-specific gene in the mouse. Yet little is known about the SNORC protein biochemical properties, and mechanistically how the gene is regulated transcriptionally in a tissue-specific manner. The goals of the present study were to shed light on those important aspects. The chondrocyte nature of *Snorc* expression was confirmed in mouse and rat tissues, in differentiated (day 7) ATDC5, and in RCS cells where it was constitutive. Topological mapping and biochemical analysis brought experimental evidences that SNORC is a type I protein carrying a chondroitin sulfate (CS) attached to serine 44. The anomalous migration of SNORC on SDS-PAGE was due to its primary polypeptide features, suggesting no additional post-translational modifications apart from the CS glycosaminoglycan. A highly conserved SOX9-binding enhancer located in intron 1 was necessary to drive transcription of *Snorc* in the mouse, rat, and human. The enhancer was active independently of orientation and whether located in a heterologous promoter or intron. Crispr-mediated inactivation of the enhancer in RCS cells caused reduction of *Snorc*. Transgenic mice carrying the intronic multimerized enhancer drove high expression of a βGeo reporter in chondrocytes, but not in the hypertrophic zone. Altogether these data confirmed the chondrocyte-specific nature of *Snorc* and revealed dependency on the intronic enhancer binding of SOX9 for transcription.

## Introduction

Bone development and formation involves a very complex series of events that need to be tightly orchestrated in a spatial and temporal manner^1,2^. A vast number of genes expressed in growth plate chondrocytes that control longitudinal growth have been identified and characterized in detail^3^. These genes encode for many different protein types that are involved in as many different processes controlling gene transcription, cellular proliferation, differentiation, shape, mineralization, adhesion, and signaling. Only few have been identified as being selectively expressed in the various phases of chondrocytes maturation and equally contributing in specific ways to the formation of a fully calcified skeleton^4^. Hence mutations in genes encoding some of those key factors controlling bone length can have catastrophic damaging incidences and lead to severe skeletal dysplasia, a group of over 450 conditions that present abnormally developed long bones and associated connective tissues^5^. The associated underlying molecular basis is, however, not always well established. Except for invasive surgical procedures, there is very few or no effective treatment currently available for diseases affecting longitudinal bone growth such as achondroplasia, the most common form of dwarfism. The growth plate is relatively avascular and this might contribute to the paucity of efficient treatment options whereby targeting the tissue of interest is most desired. However, advances in understanding the genetics behind growth plate chondrocyte regulation by the CNP/NPR3 axis have provided novel alternatives for the treatment of achondroplasia and which have proven to be efficient clinically^6,7^. There is a need, however, to expand the number of potential gene targets with comparable potential for the treatment of chondrodysplasias.

In that respect, the search for novel secreted and membrane proteins specific to bone tissue is particularly attractive: they are accessible from outside the cell, they can be exploited in several ways, either directly or indirectly, and they would ensure bone specificity. Through the use of a functional genomics tool^8^, we previously identified several candidate bone^9,10^ and tooth-specific^11^ genes. Described herein, one other gene target found and expressed specifically in chondrocytes was *3110079O15Rik* and *RGD1311447* in mice and rats, respectively. We originally retrieved its cDNA from a screen of mouse embryonic (E)14.5 limb cDNA library^8^. The human *C2ORF82* homologue was previously identified as marker of growth plate chondrocytes through RNAseq data^12^. More recently, Heinonen *et al*. named the gene *Snorc* (Small NOvel Rich in Cartilage, aka secondary ossification center associated regulator of chondrocyte maturation) and provided evidence for its expression in cartilages in mice and humans^13^. The *Snorc* mouse knockout model manifested with mild phenotypic alteration of the growth plate but without significant consequences on bone length and growth^14^. The encoded SNORC protein was predicted to be a short type I transmembrane proteoglycan whose function, biochemical properties, and gene regulation are still poorly understood. The aims of the present study were to corroborate the chondrocyte-specific nature of *Snorc* expression, further explore the biochemical and topological characteristics of the protein, and identify the mechanisms by which it is regulated at the transcriptional level.

## Results

### *Snorc* gene expression is restricted to chondrocytes

**Figure 1:**
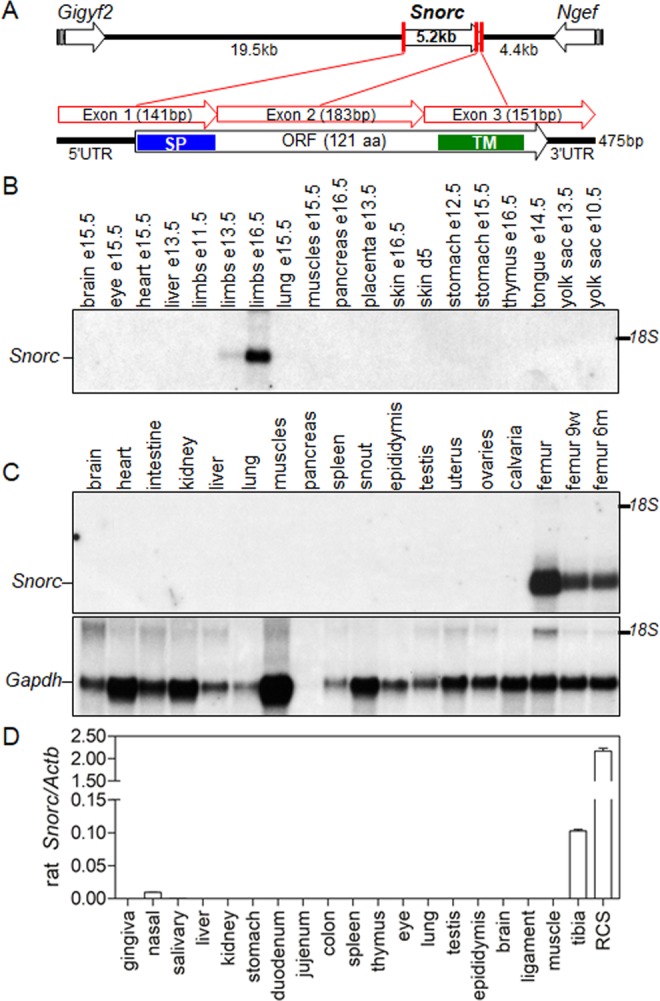
Gene expression profiling of *Snorc* in mice and rats. (**A**) Cartoon depicting the mouse *Snorc* gene, the 3 coding exons, and the mRNA with the open reading frame. Northern blot hybridization of *Snorc* in embryonic (**B**) and 3-week-old (**C**) mouse tissues (unless specified). Only limbs at E13.5 and E16.5 and femur show *Snorc* expression, declining from 3-weeks to 6-months. (**D**) Real-time qPCR assessment of the abundance of *Snorc* in 1-month-old rat tissues. Highest expression is found in tibia and rat chondrosarcoma cells (RCS). *Snorc* was also detected in the nasal septum. Values are expressed as 2e^−ΔCt^ as normalized to actin beta (*Actb*). Uncropped blots are provided in Supplemental to Fig. 1.

**Figure 2:**
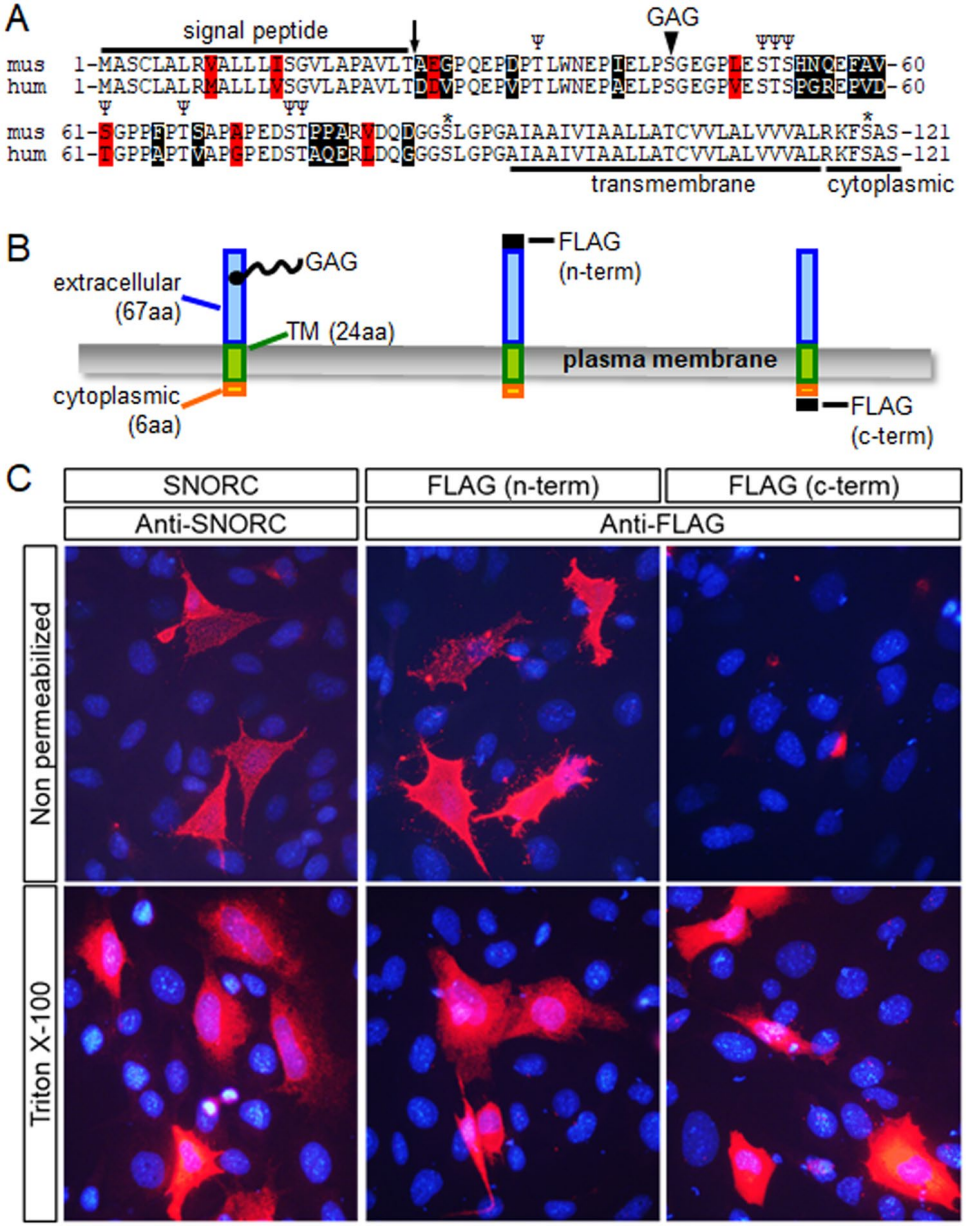
Sequence and topological configuration of SNORC. (**A**) Alignment of the human and mouse SNORC proteins showing amino acid conservation and structural features: signal peptidase predicted cleavage (downward arrow), glycosaminoglycan (GAG) attachment site at serine 44, transmembrane (TM) and intracellular domains. Predicted O-glycosylation (Ψ) and/or phosphorylation (*) sites are indicated. (**B**) Topology of SNORC at the plasma membrane and location of the inserted FLAG tag used to assess cellular distribution. (**C**) Immunofluorescence detection of untagged (left) and FLAG-tagged (right) SNORC after transient expression in ATDC5 chondrocytes. Non-permeabilized transfected cells are labeled with an antibody recognizing the extracellular portion of SNORC (residues 25-91), or when the FLAG is inserted at its N-terminus after the SP cleavage site. The intracellular C-terminally tagged SNORC is detected only after permeabilization.

**Figure 3:**
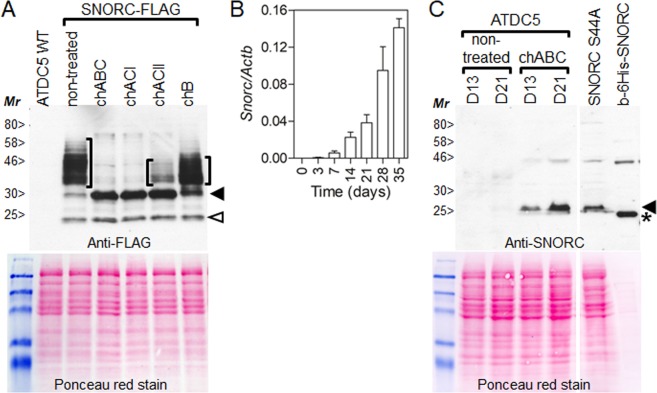
Characterization of the glycosaminoglycan composition and migration behavior of SNORC in chondrocytes. (**A**) Stable ATDC5 cells overexpressing mouse SNORC tagged at its C-terminus with the 3xFLAG epitope (SNORC-FLAG) were treated or not with the indicated chondroitinases (ch). Protein extracts were separated on 12% SDS-PAGE and blotted with anti-FLAG. In naïve cells, SNORC migrated as a smear with an apparent molecular mass ranging between 35 and 60 kDa (brackets). The smear mostly disappeared upon treatment with chABC, chACI, or chACII, and revealed a major band at ~30 kDa (closed arrowhead). Residual undigested SNORC was present with chACII and chB. An additional band was detected at around 25 kDa (open arrowhead). (**B**) Real-time qPCR analysis for the expression of *Snorc* in differentiating ATDC5 cells over the course of 35-days in culture. *Snorc* was detected at day 3, but increased steadily until day 35. Values are expressed as 2e^−ΔCt^ as normalized to actin beta (average ± SD; n = 3). (**C**) Western blot detection of endogenous SNORC in differentiated ATDC5 at day 13 and 21. The anti-SNORC antibody only detected SNORC at 25 kDa after treatment of live cells with chABC. ATDC5 expression of SNORC carrying a serine to alanine substitution at position 44 (S44A) was recognized without treatment and migrated at the same mass (arrowhead) as the bacterially expressed SNORC (asterisks). For panel C, two different western blots are presented and aligned according to molecular weight standards and ponceau red stain.

The *Snorc* gene is located on mouse chromosome 1, spans 5.2 kb, and is composed of 3 exons (Fig. 1A). The gene is well conserved across many species and the chromosomal architecture shown for the mouse is almost identical at least in the rat (9q35) and the human (2q37.1). Cloning of the mouse *Snorc* full-length cDNA revealed it is a small transcript (475nt; GenBank KM657818) containing a putative 121 amino acid (aa) open reading frame (Fig. 1A-ORF). Northern blotting was performed to assess the tissue expression pattern of *Snorc* on RNA extracted from different embryonic (Fig. 1B) and 3-week old (Fig. 1C) tissues from mice. A single transcript matching the size of the cloned full-length cDNA is expressed highly and in a restricted manner to the developing limbs (Fig. 1B), and to long bones in 3-week old mice (Fig. 1C). Levels of expression in femur are high in young mice (3-week) and decline gradually with age (Fig. 1C – compare last 3 lanes). The absence of signal in the calvaria indicated that *Snorc* is expressed in chondrocytes but not in osteoblasts. 5′RACE conducted on mRNA from day 16 differentiated ATDC5 chondrocytes led to the identification of two *Snorc* transcript variants. The first contained an upstream non-coding exon (GenBank MN970218), and the second presented with a differential splice acceptor site (AG) utilization between exons 1 and 2, leading to an in-frame deletion of the codon for alanine at position 25 (see Fig. 2A). This variant causes the coding to be shortened by 1 residue without any other significant impact on the signal peptide (SP). *Snorc* expression in 1-month old rat tissues was determined by RT-qPCR (Fig. 1D). Highest abundance was found in tibia, and about 10-times less in nasal septum. All other tissues had no detectable levels. Rat chondrosarcoma cells (RCS) had ~20-fold more *Snorc* than the whole tibia.

### SNORC protein biochemical and topological characteristics

SNORC is composed of 121 aa (or 120 aa for the splice variant) whose sequence is highly conserved among 20 different species examined (mammals, birds, amphibians, reptiles), and the alignment of the mouse and human sequences is shown in Fig. 2A. Apart from the presence of a cleavable SP and a C-terminal transmembrane domain, an in silico search for potential motifs and post-translational modifications revealed only putative O-linked glycosylation and phosphorylation sites (Fig. 2A). One other feature is the presence of a highly conserved glycosaminoglycan (GAG) attachment site on serine 44. The mature SNORC is predicted to be only 97 residues long and anchored at the cell membrane with an extracellular N-terminus (67 aa) and an intracellular C-terminus (6 aa), as schematized in Fig. 2B. In order to confirm the topological conformation of mouse SNORC at the plasma membrane, expression plasmids were constructed having a FLAG-tag inserted at the N-terminus (just past the SP cleavage site) and C-terminus (Fig. 2B). The topology of the wild type SNORC protein was verified by immunofluorescence staining after transient transfection of the respective encoding plasmids in ATDC5 chondrocytes (Fig. 2C). The untagged SNORC protein detected with an anti-SNORC antibody displayed a typical membrane-associated signal on non-permeabilized cells (Fig. 2C). Similarly, SNORC was detected extracellularly at the plasma membrane when the FLAG was present at its N-terminus (Fig. 2C middle panel) but not at its C-terminus (Fig. 2C right panel). On cells permeabilized with triton X-100, all 3 forms of SNORC were localized to an intracellular ER-like reticular pattern (Fig. 2C bottom panels). These results confirm the type I orientation of the SNORC protein at the cell surface.

### SNORC is a chondroitin sulfate proteoglycan

To determine the sugar composition of the GAG chain present on SNORC, we generated ATDC5 stably expressing the mouse SNORC tagged at its C-terminus with the FLAG tag (SNORC-FLAG). Cells were grown to confluence and live cells were treated with various bacterial chondroitinase (ch) enzymes that can selectively remove/degrade the GAG chain depending on its composition. Western blotting with the FLAG antibody showed that SNORC migrated anomalously on SDS-PAGE gels as a smear with an apparent molecular mass between 35 and 60 kDa, far exceeding its theoretical mass of 9.8 kDa (Fig. 3A, brackets). After treatment with chABC, chACI, or chACII, the smear mostly disappeared and revealed a major band at ~30 kDa (Fig. 3A, closed arrowhead). All three enzymes can only cleave and release chondroitin sulfate sugars, not heparin sulfate or dermatan sulfate, by endoglycolytic (chABC and chACI) or exoglycolytic (chACII) cleavage. In contrast to chABC and chACI, there was still residual undigested SNORC with chACII, suggesting the presence of another type of sugar. Chondroitinase B treatment, which specifically cleaves dermatan sulfate (an epimer of chondroitin sulfate) affected the migration profile only minimally indicating the presence of a low content of dermatan sulfate.

Next, we sought to confirm that endogenous SNORC also carries a chondroitin sulfate GAG side chain. The chondrocytic expression of *Snorc* was further explored in the ATDC5 cell line which has been used extensively as an *in vitro* model of chondrogenesis. ATDC5 grown in αMEM supplemented with 5% FBS and ascorbate differentiate into fully mature chondrocytes over a 3-5-week period. Expression of *Snorc* monitored by RT-qPCR (Fig. 3B), displayed a regulated temporal profile, being absent in proliferating and early differentiating cultures up to day 7, when expression starts and increases to high levels up to day 35. Differentiated ATDC5 cultures at day 13 and 21 were treated or not with chABC and protein extracts were analyzed by western blotting (Fig. 3C). When immunoblotted with the anti-SNORC antibody, SNORC was detected only on cell extracts treated with chABC as a single band at ~25 kDa which increased from day 13 to 21 (Fig. 3C arrowhead). After transient transfection in ATDC5, expression of SNORC carrying a serine 44 to alanine substitution (S44A), was readily detected without chABC treatment and migrated at the same molecular mass (~25 kDa). A bacterially expressed 6-His-tagged SNORC migrated just below the 25 kDa marker (Fig. 3C asterisk). The difference in size (about 2 kDa) between the mammalian and the bacterial SNORC, corresponds to the sugar ‘stub’ left attached to S44 after chABC treatment. These results indicated that SNORC carries a single GAG chain, containing chondroitin sulfate and a low content of dermatan sulfate. Further, the anomalous migration behavior of SNORC is possibly inherent to its amino acid composition as the S44A mutant and the bacterial form also migrated at ~25 kDa (see Fig. 3C, asterisk).

### Rat *Snorc* transcript and validation of the coding sequence

**Figure 4:**
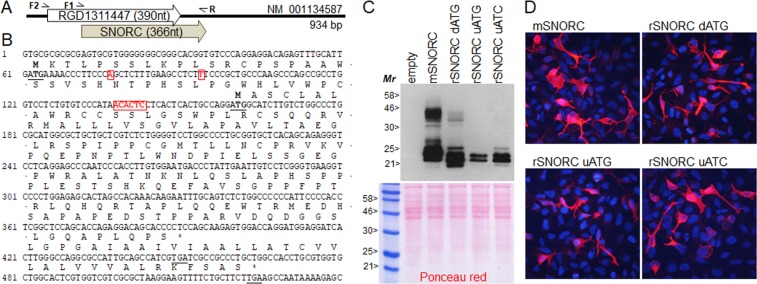
Validation of the rat *Snorc* cDNA and open reading frames (ORFs). (**A**) Schematic of the rat GenBank transcript (NM_001123587) with the currently annotated ORFs (RGD1311447 and SNORC). (**B**) Partial 5′ sequence of NM_001134587 with the 2 different ORFs. The upstream (u) and downstream (d) ATG and their respective stop codons are underlined. 5′RACE on RCS cells identified 8 transcriptional start sites (boxed red) with a main cluster at around −18 to −22 relative to dATG. The cDNAs with uATG or dATG were cloned using the indicated primers pairs (F2-R or F1-R). (**C**) Plasmids encoding mouse (m) and rat (r)SNORC were expressed in HEK293 cells and proteins analyzed by western blotting with an anti-SNORC antibody. SNORC was expressed from cDNAs with dATG only, uATG and dATG, or with the uATG mutated (uATC). (**D**) Immunofluorescence detection of SNORC on non-permeabilized HEK293 expressing the various forms as indicated.

The reference rat *Snorc* transcript in GenBank (NM_001134587) is annotated based on the amalgamation of 2 expressed sequence tags (BQ205341 and AI031033). The origin of these 2 ESTs is from E18 whole embryo and Swarm Rat Chondrosarcoma, respectively. However, the deduced ORF (RGD1311447) starts at an upstream ATG (uATG) that is not in frame with the downstream SNORC coding (Fig. 4A), does not share the same STOP codon, and encodes a totally different protein. In order to validate the authenticity of the rat *Snorc* transcript and coding sequence, we performed 5′RACE from RCS mRNA and found 8 different transcriptional start sites (from 20 randomly sequenced clones) all located downstream of the uATG (Fig. 4B red boxes). Most were located about 20 bases upstream of the SNORC ATG (dATG). A PCR approach was then used to amplify and clone the upstream and SNORC coding sequences in expression plasmids. Proteins produced after transient transfection in HEK293 cells were analyzed by western blotting using the anti-SNORC antibody (Fig. 4C). The rat SNORC protein expressed from the genuine *Snorc* cDNA (rSNORC dATG) was comparable in size and migration pattern to that of the mouse, with a high molecular smear, indicative of the GAG chain (Fig. 4C). When the uATG was present with an intact dATG, SNORC was detected to a lower extent, suggesting a preference towards usage of the dATG. Mutation of uATG (uATC) caused a slight increase in abundance of rat SNORC compared to the uATG-containing cDNA. Immunofluorescence staining for SNORC on intact non-permeabilized HEK293 cells also revealed the characteristic membrane signal for all forms tested (Fig. 4D). These data suggest that in the rat, the NM_001134587 transcript encodes the rat SNORC protein.

### Transcriptional regulation of the *Snorc* gene

**Figure 5:**
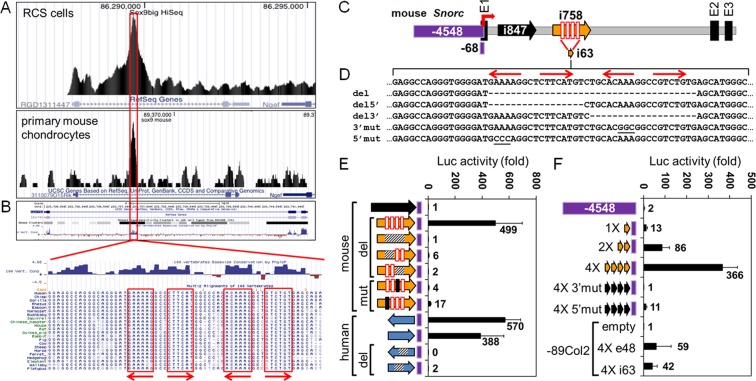
Transcriptional regulation of the mouse *Snorc* gene. (**A**) ChIP-seq data indicating the region of SOX9 binding on RCS cells and mouse chondrocytes. (**B**) The rat and mouse SOX9-peaks center on a highly conserved region of *Snorc* intron 1 with sequences having similarity to the SOX9 binding sites (inverted repeats, boxed). (**C**) Cartoon depicting the various segments of the mouse *Snorc* gene tested for their transcriptional activity in luciferase reporter assays (E1, E2, E3 denote exons). The regions indicated are defined in bp; upstream promoter -4548; 5′UTR -68; intron 1 sequences i847, i758, and i63. (**D**) Sequence of intron i63 with the 2 putative bipartite SOX9 binding sites indicated as red arrows and red boxes in i758 (**C**). Three deletions and 2-point mutants (underlined nucleotides) were introduced as indicated and cloned upstream of the *Snorc* -68 5′UTR segment into the pGL4 basic luciferase (Luc) reporter. E, F) Plasmids were transfected in RCS cells and Luc activity measured 24 h later. All values are expressed as fold increase relative to the control -68 devoid of other elements. (**E**) Except for i847, all constructs tested were in the i758 context. The human i427 segment activity is shown in blue arrows at the bottom in the antisense and sense orientations. (**F**) The -4548 had minimal activity while the monomeric and multimeric forms of i63 were increasingly active. The 4X i63 was also tested within the collagen type 2 promoter reporter -89Col2^16^ and compared to the Col2 exon 1 (e48) multimerized SOX9 enhancer.

To explore the mechanisms by which the mouse *Snorc* gene is regulated at the transcriptional level, we conducted a series of complementary experiments based on various assays in rat chondrosarcoma cells (RCS), in which rat *Snorc* was found constitutively expressed (Fig. 1D). The first step consisted at identifying which mouse *Snorc* gene regulatory regions would drive expression of luciferase reporter constructs in RCS (Fig. 5). A 4.5 kb DNA segment upstream of the *Snorc* transcriptional start site cloned into the pGL4-basic luciferase was mostly inactive (Fig. 5F), indicating that the regulatory regions were not located in the upstream promoter region. Chromatin immunoprecipitation sequencing (ChIP-seq) experiments conducted in the context of a previous publication^15^, aimed to identify SOX9 binding sites throughout the mouse and rat genomes, revealed a conserved peak in intron 1 of the *Snorc* gene in both mouse primary chondrocytes and RCS cells (Fig. 5A). Analysis of the genomic sequence around the peak center revealed a highest conservation across species, with two sites bearing high resemblance with SOX9 binding sites, configured in a head to head configuration (Fig. 5B, red boxes and arrows).

Two arbitrary segments of 847nt and 758nt (i847 and i758) of the mouse intron 1 were amplified and cloned into the pGL4-basic luciferase reporter just upstream of a minimal promoter corresponding to 68nt of the mouse *Snorc* 5′UTR (Fig. 5C). The i758 corresponded to the ChIP-seq peak, while the i847 was tested as it also contained predicted SRY sites (Fig. 5C top). Transient transfection in RCS cells yielded a robust response only for i758 (Fig. 5E), confirming it contained bona fide regulatory elements. To test whether the activity was also conserved in the human, a 427nt portion (i427) of the human *SNORC* enhancer was also cloned and tested for activity in RCS (Fig. 5E). Both the antisense and sense configuration of human i427 gave high activity, whereas i427 having deletion of the SOX binding motif abrogated the activity (Fig. 5E – blue arrows). A series of internal deletions (Fig. 5D) within the i758 region targeting the 3′ site, 5′ site, or both, abolished all activity (Fig. 5E), pointing to the importance of the central SOX9-like binding sites. The luciferase reporters harboring point mutations introduced in the 5′ or 3′ sites only gave negligible residual activity (Fig. 5E). Deletion of the corresponding 39 bp SOX9 binding site within the human i427 construct was inactive (Fig. 5E). A single shorter 63 bp segment (i63) covering the central SOX9 binding sites of i758 also could drive efficient luciferase readouts (13-fold) (Fig. 5F). Multimeric copies of i63 (dimer and tetramer) gave synergistic increments of 86- and 366-fold respectively. Again, point mutations introduced within the 3′ and 5′ sites of the tetrameric i63 segment rendered the reporter almost inactive (1.4- and 11-fold). As a comparison, a 4-copy multimer of the 48 bp collagen type 2 (Col2) enhancer cloned upstream of its own -89bp minimal promoter (-89Col2) into the pGL2-basic luciferase reporter drove significant but comparable activity to the i63 when tested in the same Col2 backbone plasmid (Fig. 5F). The mouse *Snorc* -4.5kb-Luc reporter remained to background levels in HEK293 cells even when co-transfected with a SOX9 expression plasmid (supplemental Fig. 1), suggesting the absence of functional SOX9 binding sites in that promoter region.

### SOX9 binds to the intronic region of *Snorc*

**Figure 6:**
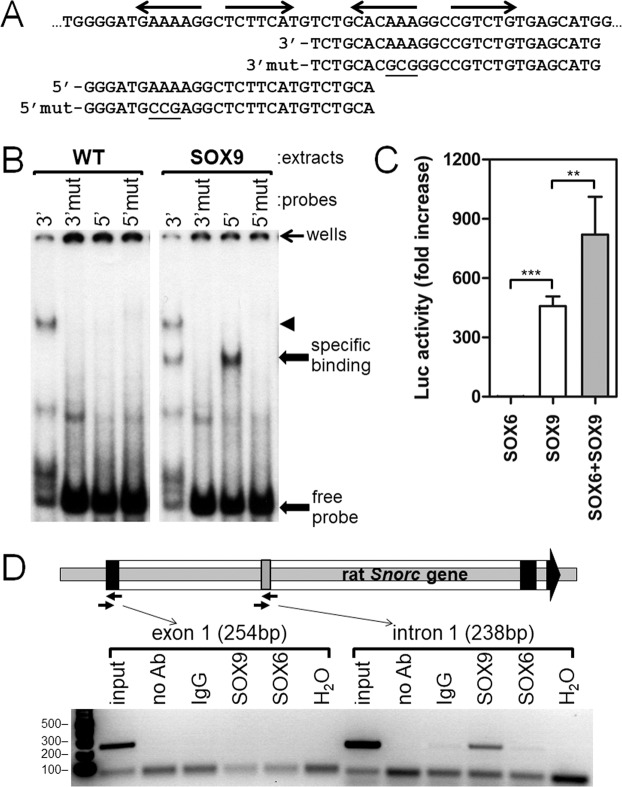
SOX9 binds the *Snorc* intronic enhancer and regulates transcriptional activity. (**A**) Oligonucleotide sequences of the mouse *Snorc* used for EMSA. (**B**) EMSA using nuclear extracts prepared from wild type (WT) or HEK293 expressing hSOX9. SOX9 extract caused retardation of the 3′ and 5′ probes (specific binding), but not the mutant probes. The arrowheads indicate nonspecific binding on the 3′ probe. (**C**) Co-transfection experiments in HEK293 with the luciferase reporter containing the multimerized intronic segment (4xi63) and expression plasmids for SOX6 and SOX9. Luc activity was measured 24 h later and all values are expressed as fold increase relative to the control -68 plasmid. Combination of SOX6 and SOX9 produced greater activity than SOX9 alone, whereas SOX6 was inactive (average ± SD; n = 3; **p < 0.01; ***p < 0.001). (**D**) ChIP assay performed on RCS cells confirms that SOX9 occupies the rat intronic enhancer *in vivo*. Absence of antibodies (no Ab) or use of a non-immune rabbit IgG served as negative controls. An antibody to SOX6 did not yield any specific signal over the negative controls. Uncropped images of panels B and D are provided in Supplemental to Fig. 6.

In order to determine whether SOX9 can bind *in vitro* to the intronic region of the mouse *Snorc* gene, electrophoretic mobility shift assay (EMSA) and chromatin immunoprecipitation (ChIP) were performed. Two oligonucleotide probes were designed to cover the 5′ and 3′ binding sites (Fig. 6A). The same mutations of 3nt that abolished the luciferase reporter activity (Fig. 5D–F) were introduced in the oligonucleotides (3′mut and 5′mut) (Fig. 6A). The radiolabeled probes were incubated with nuclear extracts from wild type or SOX9 expressing HEK293 cells and analyzed by EMSA (Fig. 6B). Both the 3′ and 5′ probes generated a SOX9 specific retarded band (Fig. 6B). The 4X i63-Luc construct was next tested by co-transfection in HEK293 cells to determine the transcriptional effects of SOX6 and SOX9, alone or in combination. SOX9 triggered a very robust increase in Luc activity compared to SOX6, which did not cause any significant activation over the control empty plasmid (Fig. 6C). However, simultaneous expression of SOX6 and SOX9 induced a synergistic fold increase in activity. ChIP experiments were next performed on chromatin from RCS cells using 2 different primer sets covering regions of exon 1 and i758 (Fig. 6D). ChIP with a SOX9 antibody yielded the predicted 238 bp amplification product from intron 1, but not from exon 1 (Fig. 6D). Control IgG and SOX6 antibodies did not produce any significant amplification products. As a positive control for the ChIP, both SOX9 and SOX6 were found on the aggrecan gene enhancer (supplemental Fig. 2) that is located about 10 kb upstream of exon 1 as previously described by others^17,18^.These results suggest that SOX9 can bind to both sites of the intronic region *in vitro*, activate transcription in a heterologous system, and occupy the rat intron under baseline conditions in RCS cells.

### The *Snorc* intronic enhancer can drive activity of a heterologous βGeo reporter construct in HEK293 and RCS

**Figure 7:**
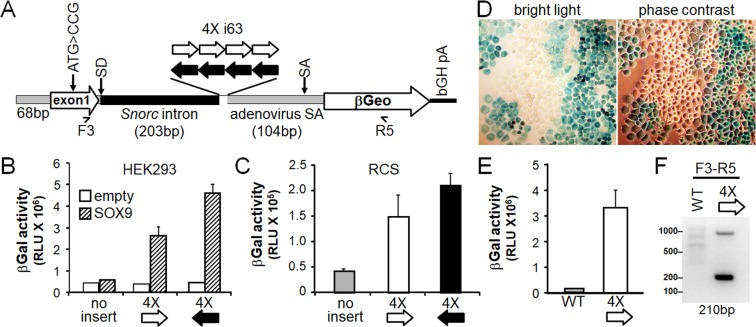
The *Snorc* enhancer drives expression of a heterologous βGeo reporter. (**A**) Cartoon depicting the features of the *Snorc*-βGeo construct. A mouse *Snorc* gene fragment of 431 bp was subcloned into the βGeo reporter containing the adenovirus splice acceptor (SA). The 4X i63 enhancer was inserted in the sense and antisense orientation in the chimeric intron as illustrated. To ensure translation from the βGeo coding, the endogenous *Snorc* ATG was mutated. (**B,C**) The constructs were tested for beta-galactosidase (βGal) activity 24 h after transient transfection in HEK293 (**B**) and RCS (**C**) cells (average ± SD; n = 3). In HEK293, co-transfection with SOX9 was required to obtain activation, whereas it was constitutively active in RCS. (**D**) A pool of RCS cells with stable integration of the construct with the sense *Snorc* enhancer expressed variable levels as determined by X-gal staining of cell monolayer. The construct without the 4x i63 enhancer did not give rise to any G418 resistant cells. (**E**) Cellular extracts from stable RCS pools with the sense 4X i63-βGeo had high levels of βGal activity. (**F**) Adequate splicing of the intronic sequence was determined by RT-PCR using primers located within exon 1 and βGeo. The uncropped image of panel F is provided in Supplemental to Fig. 7.

The enhancer activity of the mouse *Snorc* gene was tested in a heterologous beta-galactosidase-neomycin fusion (βGeo) reporter plasmid. The βGeo reporter was engineered with 431 bp of the mouse *Snorc* gene, comprising the proximal promoter (87 bp), the entire exon 1 with ATG mutated to CCG (141 bp), and 203 bp of intron 1 (Fig. 7A). The 4X i63 multimer was cloned in the sense and antisense orientations between the *Snorc* and the adenovirus introns, each providing the splice acceptor (SA) and donor (SD) sites (Fig. 7A). The beta-galactosidase (βGal) activity was measured after transient transfection in HEK293 (Fig. 7B) and RCS (Fig. 7C) cells and compared to the same construct lacking the 4X i63 intronic sequence. In HEK293, the βGeo reporters all gave baseline activity in the absence of SOX9 (Fig. 7B). Upon co-transfection with SOX9, however, the 4X i63 constructs gave significant 5- (sense) to 4.5-fold (antisense) increase in βGal activity as compared to the control (Fig. 7B). In naïve RCS cells, the sense and antisense 4X i63 gave significant (3.8- and 5-fold) increases in βGal over the empty construct (Fig. 7C). Next, the empty βGeo reporter construct and the one carrying the sense 4X i63 were stably transfected in RCS and a pool of cells resistant to neomycin was obtained only for the latter, demonstrating expression of the resistance βGeo cassette (Fig. 7D). The RCS cells stably expressing the 4X i63 showed strong but non-uniform staining with X-gal (Fig. 7D), with some cells remaining unstained. Cell extracts contained considerable βGal activity (Fig. 7E) which was about an order of magnitude higher than that detected in the transient expression (Fig. 7C). RT-PCR also confirmed that the transcript from the βGeo construct was spliced efficiently and correctly as assessed with primers contained within *Snorc* exon 1 and βGeo, giving rise to the expected 210 bp product (Fig. 7F). These data indicate that the *Snorc* SOX9 enhancer can efficiently drive expression of βGeo irrespective of orientation when cloned within a heterologous chimeric intron.

### Crispr-induced mutation of the SOX9 intronic enhancer reduces endogenous *Snorc* expression in RCS

**Figure 8:**
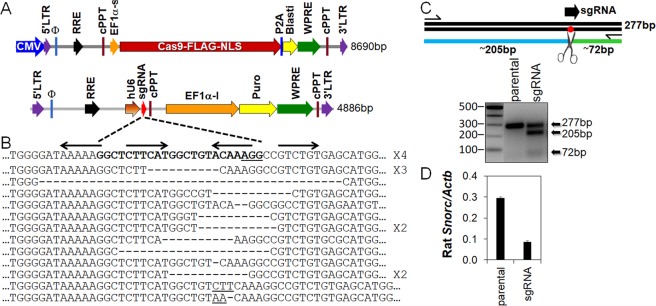
Targeting the *Snorc* intronic enhancer in RCS reduces endogenous expression. (**A**) Stable RCS cells expressing the humanized Cas9 enzyme and the rat *Snorc* enhancer guide RNA (sgRNA) were generated by lentiviruses with the indicated plasmids. (**B**) The *Snorc* sgRNA (bolded) was designed to cover part of the 5′ and 3′ SOX9 binding sites. Genomic DNA was extracted from the pool of stably expressing RCS, amplified with flanking primers, cloned and sequenced (number of clones indicated at right). Sequencing revealed a wide array of events, with deletions of 2 bp up to 30 bp, and smaller 2-3 bp insertions (bottom rows). Some clones were unaltered and matched the wild type rat sequence (upper row). (**C**) The extent of targeting was assessed with the Surveyor kit. Similar to the sequencing data, about 50% of cells presented with indels. (**D**) RT-qPCR monitoring of the endogenous rat *Snorc* transcript indicated about 60% reduction in its expression levels. The uncropped image of panel C is presented in Supplemental to Fig. 8.

In order to delineate if the intronic enhancer region is essential for normal endogenous rat *Snorc* expression, we used the Crispr technology to introduce indel mutations in the RCS cells. For this purpose, we generated stable pools of RCS expressing the Cas9 enzyme and a guide RNA targeting the SOX9 enhancer binding site as depicted in the cartoon of Fig. 8A. This was done by consecutive lentiviral-mediated transgene delivery of both constructs and selection with blasticidin and puromycin. Genomic DNA from the ensuing pool of resistant cells was extracted and PCR-amplified using primers flanking the intronic enhancer segment. The products obtained were sub-cloned in the pBluescript plasmid and 20 randomly picked clones were sequenced (Fig. 8B). Several clones had different indels as indicated: some were missing the entire or part of the SOX9 binding sites, others were wild type (top row) or had mutations with possibly minimal impact on the enhancer, indicating a heterogeneity in the efficacy of the guide. The surveyor assay conducted on gDNA isolated from the parental Cas9 or sgRNA pool indicated a 50% efficacy of targeting (Fig. 8C), in line with the sequencing data. Despite this partial targeting efficiency, RT-qPCR analysis for the rat *Snorc* indicated that expression was reduced by 60% (Fig. 8D). These results indicate that the intronic enhancer is necessary to drive *Snorc* expression in RCS.

### The *Snorc* intronic enhancer can drive βGal expression in chondrocytes in transgenic mice

**Figure 9:**
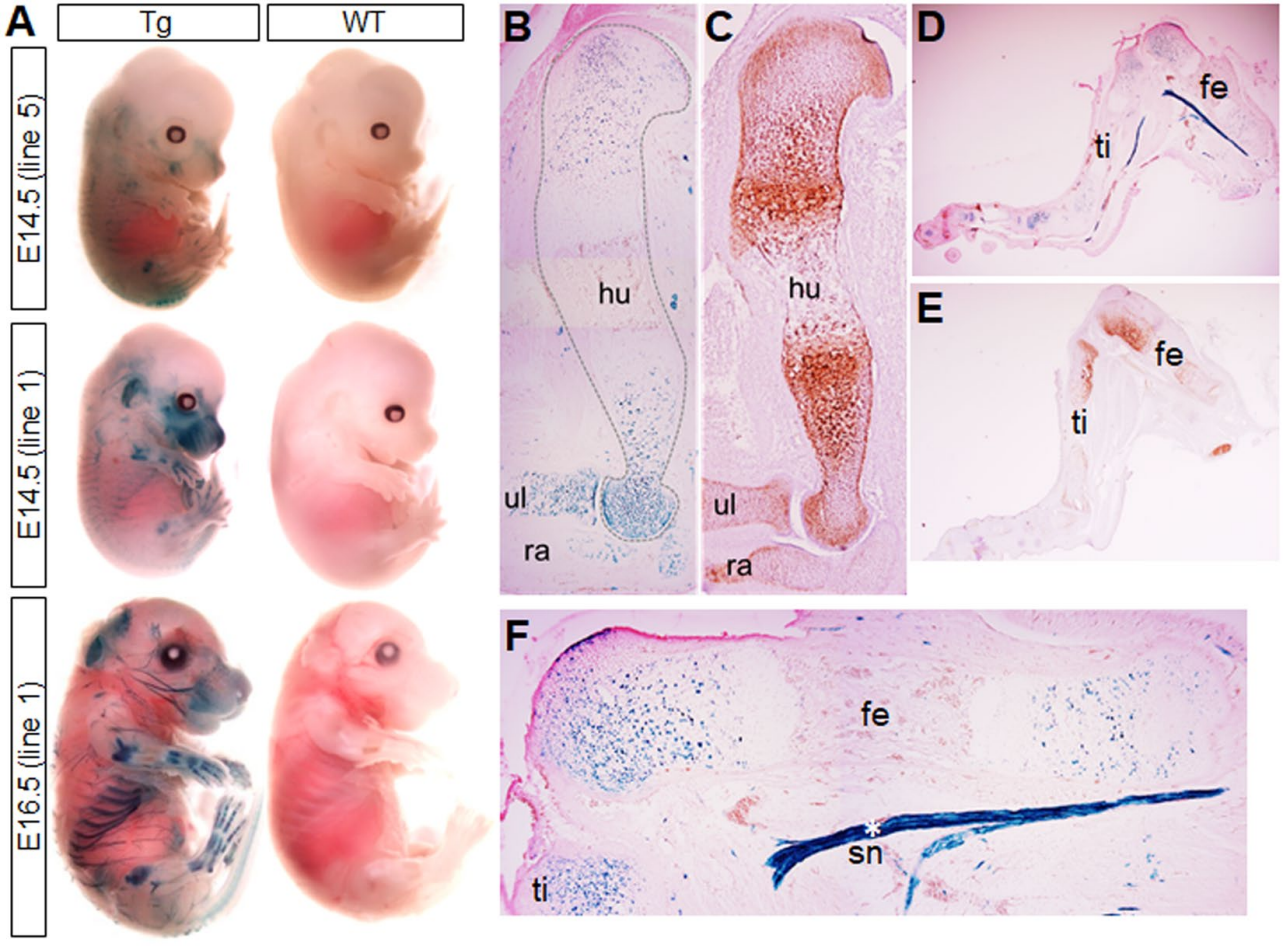
The *Snorc*-βGeo construct drives βGal expression in chondrocytes *in vivo* in mice. The *Snorc*-βGeo construct was used for pro-nuclear injection to generate 2 transgenic (Tg) mouse lines. (**A**) Two lines (5 and 1) were found to express detectable X-gal after whole mount staining at E14.5 (2 h staining). Both lines presented with X-gal in most of the developing cartilaginous tissues (rib cage, long bones, digits, face, ear). By E16.5, the X-gal stain was prominent in line 1, with most of the cartilaginous elements being stained heavily. Other tissues also turned blue, especially the nerve bundles in the jaws (trigeminal), ribs region, and legs (sciatic). Control wild type (WT) littermates did not present any X-gal staining at both stages. (**B**,**D**) Fore- and hind-limbs at E14.5 (**B**) and E16.5 (**D**) were processed for cryosectionning and X-gal staining. The chondrocytes of the growth plate were positive for βGal. (**F**) Magnified image of panel D showing the E16.5 femur with adjacent sciatic nerve (*). (**C**,**E**) Immunohistochemical detection of SNORC on consecutive tissue sections for the humerus at E14.5 (**C**) and leg at E16.5 (**E**). Abbreviations: humerus (hu), femur (fe), tibia (ti), sciatic nerve (sn), ulna (ul); radius (ra).

To determine whether the *Snorc* enhancer could drive expression in chondrocytes in mice, the βGeo construct detailed in Fig. 7A was used to generate transgenic mice. The DNA segment was excised from the remaining plasmidic sequence, gel purified, and used for pro-nuclear injections. Founders carrying 4- (line 5) and 8-copies (line 1) of the transgene were mated and embryos from staged pregnant females were collected for analysis by whole mount X-gal staining (Fig. 9). In E14.5 embryos, both lines displayed restricted X-gal coloration in most of the cartilage elements, whereas the non-transgenic littermates showed no staining (Fig. 9A). The X-gal staining in line 1 was more robust than in line 5, in which X-gal was detected in the frontal head, ear, cartilage portion of the rib cage, most long bone of arms and legs (Fig. 9A). The staining was more pronounced by E16.5 with some staining appearing at the interparietal bone structure at the back of the head (Fig. 9A). In both lines staining was also clearly visible in nerves, possibly corresponding to the trigeminal in the head and sciatic in the legs (Fig. 9A). X-gal staining of cryosections of the front and hind limbs at E14.5 (Figs. 9B) and E16.5 (Fig. 9D,F), respectively, showed intense staining of the growth plate chondrocytes, mostly the resting and proliferative zones, regions which also stained positively for endogenous SNORC by immunohistochemistry (Fig. 9C,E). Again, nerve bundles of the sciatic nerve and other discrete area were stained with X-gal (Fig. 9D,F), but did not for SNORC protein.

## Discussion

The long bones are formed through an endochondral ossification process, whereby a cartilage template is first deposited by chondrocytes and onto which bone further builds^1,2^. This mechanism of bone growth requires an array of molecules which tightly govern a complex series of spatio-temporal events^3^. Disruption of any one of the critical steps regulating the chondrocyte proliferation, differentiation, and late stage hypertrophy can lead to a vast number of skeletal dysplasia^5^. Very few genes have been studied for which expression was found to be chondrocyte-specific and *Snorc* likely represent one such tissue-specific gene^13^ but little is known about its function. The present study was undertaken to further characterize the molecular aspects of the protein biochemical properties and gene transcriptional regulation. We have confirmed that *Snorc* expression in rodent tissues is restricted to cartilaginous structures, either permanent like the nasal septum or transient like the long bone growth plates. *Snorc* was found to be expressed in mouse and rat chondrocyte cell lines. We provided a comprehensive biochemical analysis looking at the topological conformation of SNORC, its GAG composition, and migration behavior on SDS-PAGE. The rat cDNA sequence was cloned and used to confirm it encodes the SNORC protein, despite conflicting in silico information. Further, we identified the primary regulatory elements necessary to drive *Snorc* expression in chondrocytes, and unequivocally identify SOX9 as a primary and important regulator of an intronic enhancer.

Heinonen *et al*. first reported the description of *Snorc* and its basic details^13^. They showed it is a proteoglycan expressed in chondrocytes by *in situ* hybridization and immunohistochemistry, and that it can be regulated by BMP4 *in vitro*. We have now complemented those initial observations by corroborating the expression profiling of *Snorc* in mouse and extending it to rat tissues. In both species, *Snorc* is highly restricted to chondrocyte-containing tissues (as early as E13.5 in the mouse) and expressed in chondrocyte cell lines (mouse ATDC5 and rat RCS). ATDC5 cells represent a widely used model to study chondrogenesis in cultures^19–21^. In ATDC5, *Snorc* was found to be expressed to significant levels only after a week in culture but increasingly expressed in late differentiation. This would suggest that *Snorc* is not needed during early differentiation and that its function pertains more to a mature chondrocyte. In support of this assumption, we found that *Snorc* is constitutively expressed in RCS, which are derived from rat chondrosarcoma and display morphological and genetic characteristics of permanent chondrocytes^22,23^. As such RCS represented a unique system to further study the mechanisms regulating transcriptional regulation of the *Snorc* gene as discussed further below. Other published studies, performing broader gene expression profiling by microarrays or RNAseq, have also anecdotally reported *Snorc* expression in chondrocytes in other species. For instance, *SNORC* (*C2ORF82*) was listed as a highly chondrocyte-enriched gene in human growth plate chondrocytes^24^, and detected in human osteoarthritis cartilage tissue^25^. It was also found significantly upregulated in human^26,27^ or equine^28^ cultured mesenchymal cells differentiated into chondrocytes. We have also cloned the human *SNORC* cDNA (GenBank MN974270) from the human osteosarcoma U2-OS cells line, consistent with its reported expression in osteosarcoma^29^. Another broad gene expression study found *Snorc* to be among the 10 top most enriched genes expressed in enthesis relative to tendon in the pig^30^. Feregrino *et al*.^31^, while cataloging genes expressed the chicken embryonic distal limb by single cell RNAseq, reported that *C9H2ORF82* (*Snorc*) clustered with *Col9a1* and *Acan*. Altogether, these results point to the emergence that *Snorc* represents a specific marker of differentiated chondrocytes. Despite those findings, some caution must be exercised as still other unappreciated expression sites for *Snorc* could have implications. For instance, *SNORC* expressed sequence tags were identified from brain tissues in humans which could bear as important functional significance, in light of more recently reported association studies between *SNORC* and brain-related disorders (schizophrenia, attention-deficit/hyperactivity)^32,33^.

At the biochemical level, we provided experimental evidence for the predicted topological conformation of the SNORC protein that was originally suggested^13^. Tagging and immunofluorescence results showed that SNORC adopted a type I configuration with a single transmembrane passage, and carry a single chondroitin sulfate GAG side chain attached to serine 44 on its extracellular domain. Detailed analysis of GAG chain by chondroitinase treatments suggested it is mainly composed of chondroitin sulfate (CS), although this does seem to be entirely homogenous. Glycosaminoglycans (GAGs) are extended chains of linear repeating disaccharides composed of either N-acetyl galactosamine or N-acetyl glucosamine and glucuronic acid. These sugars are added post-translationally in the Golgi to serine residues onto core proteins moiety and can be further extensively modified in subsequent steps that lead to at least sulfation and epimerization. The two major types of GAGs are called heparan sulfate and chondroitin sulfate, depending on their sugar compositions and degree of modifications. Chondrocytes manufacture several different proteoglycans that are either secreted (aggrecan, decorin, biglycan, epiphycan, perlecan), transmembrane (syndecan, CD44, TGFbeta3 receptor, neuropilin, CSPG4/NG2) or membrane-anchored (glypican)^34^. The highly net negative charge conferred by GAGs, allows water retention and consequently resistance to compression load of articular cartilage. Beyond hydration of the tissue, however, the GAGs have been shown to interact with and control the activity of many other secreted factors (IHH, FGF, BMP antagonists) equally important for growth plate development. Interaction of these factors with selected proteoglycans can either limit their diffusion, modulate their presentation to cell surface receptors, and/or protect them from degradation^35–39^. It should be mentioned that the GAG chain(s) in a given protein is often heterogeneous in nature and can change with respect to age or diseased status^40^. In that respect, SNORC present similarities with a number of other type I proteins with important function in growth plate chondrocytes. Syndecans and versicans are comparable size proteins that have been involved in regulating chondrocyte cell activity, such as effecting directly through attachment, migration and proliferation. They have also been described to mediate binding and presentation of many different growth factors and mediate their availability and presentation to their respective receptors on the cell surface^41^. Most harbor 1 or 2 heparan-sulfate GAG chains, which is the preferred binding moieties for FGFs, BMPs, hedgehog, WNT. Biochemical analysis revealed that the GAG chain attached to SNORC was mainly composed of CS, not the preferred binding site for FGFs^42,43^. It is presently unknown whether the function of SNORC is solely based on its GAG and whether it can mediate any direct or indirect intracellular signalling. In agreement with our current analysis, Heinonen *et al*. have shown that recombinant SNORC could bind FGF2 *in vitro*, but the interaction was mediated by the protein ectodomain, not by the GAG chain^14^. Although the SNORC ectodomain could negatively affect FGF2-induced proliferation of C3H cells^14^, it remains to be established whether the function of SNORC in vivo would be to convey FGF signalling. Indeed, the knockout mouse model for *Snorc* presented only with a mild phenotype in post-natal stages^14^. Knockout mice at 10 and 22 days, primarily displayed an enlarged growth plate and a smaller secondary ossification center relative to wild type littermates^14^. The growth plate alterations did not however impact the overall long bone length and mineralization. At the molecular level, expression for *Mmp13* was decreased, whereas that of *Ihh* was increased. This was associated with a change in shape of columnar chondrocytes being more rounded. The mechanism of SNORC action in vivo remains to be further elucidated.

Although the presence of a single GAG chain on SNORC contributed to its aberrant migration on SDS-PAGE, other factors may be involved. SNORC producing cells treated with chABC resulted in a protein with an excess of 19 kDa relative to its expected mass. The S44A mutant migrated to almost exactly the same mass, indicating that the core protein structure also contributed to the observed anomalous mobility shift. The fact that the bacterially expressed SNORC migrated similarly to the mammalian form also suggested that no major post-translational modifications (O-glycosylation) occurred on the protein. The abnormal migration on SDS-PAGE is not uncommon for transmembrane proteins and has been explained in part to be due to incomplete SDS titration of hydrophobic segments^44,45^. However, bacterial expression of a form of SNORC lacking the transmembrane domain (that was used for generation of the antibody) also migrated slowly (data not shown). In silico structural analysis of the SNORC polypeptide indicated that it is intrinsically disordered and that could partly explain this observation, a feature common to syndecans^46^. The net charge carried by the extracellular domain of SNORC at neutral pH (−11) may also have influenced migration to some extent due to the high content (18%) of aspartic and glutamic acid (D/E) residues. It has been documented that acidic proteins generally migrate more slowly due to negative charge repulsion with SDS^47,48^. However, use of a mathematical formula put forward to predict the molecular mass shift based on the percentage of D/E residues in a polypeptide^48^, only predicted a change in mass for SNORC by a mere 1.2 kDa, which did not match that of the observed change (+19 kDa). SNORC therefore represents one of numerous examples of proteins migrating anomalously on SDS-PAGE.

Information deposited in publicly available databases (GenBank and others) pertaining to gene expression and products can sometimes be misleading and require experimental validation. This was the case for the rat *Snorc* sequence (NM_001134587) which was derived from 2 separate sequences, and assigned a different protein coding than SNORC. Upon further inspection, several empirical and experimental data would point to an erroneous annotation. First, our 5′RACE experiments did not allow identifying mRNA that included the uATG site in both the rat and mouse. Most transcripts clustered at position between −22 to −16 relative to the dATG. Second, although the designated upstream initiator ATG (uATG) is conserved across 10 different species, its context (TTGATGA, with the ATG underlined) is not predicted to conform to a strong initiating methionine based on the Kozak rule which calls for a purine at position −3 and/or a guanosine at position +1 (RCCATGG)^49^. Further, the rat would be the only species having a significant length contiguous ORF starting at uATG (129 residues), whereas STOP codons are present shortly after in many other species giving rise to very short 16 residues ORF. In fact, the dATG representing the *Snorc* translation start site, is also conserved in all species (GCCAGGATGG) and obeys to a strong Kozak sequence. In silico prediction of the rat *Snorc* translation start site using NetStart (http://www.cbs.dtu.dk/services/NetStart/) yielded scores of 0.299 and 0.868 (cut off of 0.5) for the uATG and dATG, respectively, favoring the dATG SNORC translation site. Third, expression studies performed herein indicated that even in the presence of a longer 5′UTR extending upstream to the uATG, SNORC was still produced, albeit less than when uATG was mutated, thereby suggesting that the uATG has no major impacts on SNORC synthesis. Altogether these data suggest that rat SNORC has the same coding sequence as the mouse. However, an appreciation of more complex rules governing the mechanisms of translation initiation at upstream start sites has recently emerged^50,51^, and these could represents additional mechanisms impacting on ATG utilization and SNORC production.

ChIP-seq is a powerful technique to probe for unbiased binding sites for a given transcription factor throughout the genome^52^. The *Snorc* intronic element was originally identified by 2 co-authors of the current study using such a ChIP-seq approach in mouse primary chondrocytes and RCS cells^15^. Using ChIP-Seq, other studies^18,53^ also found SOX9 binding to the same mouse intronic region we identified here. Through a number of different experiments, we have now shown that expression of *Snorc* is mediated mainly through this highly conserved SOX9 enhancer within intron 1. The -4.5 kb promoter region of *Snorc* was mainly silent in RCS cells indicating that other endogenous transcription factors could not bind and activate in this context. However, it cannot be excluded at this point that other regulatory regions, lying outside the sequences we analyzed so far, could participate to *Snorc* expression in chondrocytes. Along that line, epigenetic regulation (CpG demethylation) of the *SNORC* promoter region has recently been shown to correlate with chondrogenic differentiation of human bone marrow stromal cells^54^. Transient transfection of Luc reporter constructs containing the mouse or human intronic segment could elicit very high activity in SOX9 + RCS cells, which were used as a surrogate system with endogenous expression of *Snorc* and SOX9^22,23^. We provided further confirmation of the activity of this enhancer by showing that SOX9 can bind both diads *in vitro* by EMSA, and that the region was constitutively occupied by SOX9 in vivo in RCS as determined by ChIP. The *Snorc* intronic bi-partite sequence was found to correspond well to the previously reported SOX9 DNA motif in cartilage responsive gene. The motif typically feature two binding sites oriented head-to-head and separated by 3-4 nucleotides ((A/T)(A/T)CAA(A/T)GN_3-4_C(A/T)TTG(A/T)(A/T))^17,55,56^. However, the preferred SOX6 de novo motif was reported to be different than the SOX9 and more variable being A-rich^18^. In accordance to the strict definition of an enhancer, the *Snorc* enhancer was active independent of the orientation and whether it was located upstream of a transcriptional start site (Luc), or within an intronic location in a heterologous reporter. When it was tested *in vitro*, its activity was inducible by SOX9 in non expressing HEK293, but constitutive in expressing RCS cells. In non expressing HEK293 cells, we also showed that co-transfection with SOX9 but not SOX6 could activate expression. SOX6 does not contain an activation domain and was not expected to be active alone^57^. In HEK293, however, the combination of both SOX9 and SOX6 elicited a synergistic response, which suggested the binding and interaction of both transcription factors as described previously^57^. We could not find evidence, however, that SOX6 was bound to the enhancer by ChIP in RCS and further experiments will be required to elucidate this conundrum, especially in light that a ChIP-seq SOX6 enhancer was identified in the *Snorc* intron region^18^. Deletion of the enhancer element by CRISPR also significantly reduced expression levels of *Snorc* in RCS, indicating that it is essential for maximal expression. When tested in transgenic mice, the intronic *Snorc* enhancer was also able to confer robust expression of the βGeo reporter in chondrocytes. It should be noted, however, that X-gal staining in our model did not fully mirror SNORC protein domain, which extended up to the pre-hypertrophic/hypertrophic regions (Fig. 9) and as was previously reported by in situ hybridization^13^. This could indicate that our transgenic fragment lacked some other regulatory elements required to recapitulate *Snorc* expression. Indeed, in the LacZ knockin model developed by Heinonen *et al*.^14^, X-gal staining extended to the hypertrophic zone, thus suggesting a stricter transcriptional control than in our model. This LacZ knockin would have had left the intronic enhancer intact, LacZ having been inserted about 2.5 kb further downstream within the intron. In addition, we observed ectopic X-gal staining in nerves that do not express SNORC. The sciatic nerve and other neuronal tissues are known to express significant levels of SOX9^58–60^, which could have contributed to activate the *Snorc* construct within the transgenic context we used. Our data describing the importance of the *Snorc* intron enhancer are additionally supported by, and pointing to conservation in other species, a previous study reporting the ChIP-seq SOX9 peak in chicken chondrocytes within *Snorc* intron 1, although the exact location could not be inferred from the published data^61^. Lastly, inactivation (knock-out) of the *Sox9* gene in mice also resulted in a dramatic reduction of *Snorc* expression level^15,18^, further suggesting that *Snorc* is regulated by SOX9 *in vivo*.

## Materials and Methods

### Expression plasmids

All custom plasmids construction was commonly generated by PCR amplification. The primer sequences and purpose are listed in the Supplemental Table 1. Routine PCR reactions (25ul) were conducted with the high fidelity Phusion DNA polymerase (NEB) as follows: 1X Phusion HF buffer, 0.3ul gDNA, 0.2 mM dNTPs, 0.5uM of each reverse and forward primers, 0.5 units of Phusion DNA polymerase. The cycling conditions were 98°C/2 min; 98°C/10 sec; 58°C/25 sec; 72°C/10 sec/kb for 27 cycles. Products were separated by agarose gel electrophoresis and purified on Minelute gel extraction columns (QIAGEN). Products were cloned into the destination plasmid for expression (CMV-based pCDNA3.1 or pGL4.10 basic for luciferase reporter). All expression plasmids are listed in Supplemental Table 2. Sanger sequencing on an Applied Biosystems 3730xl DNA Analyzer was done at the McGill University Genome Quebec Innovation Centre to verify the identity of cloned sequences. After confirmation, respective plasmids were prepared using the Midiprep Qiafilter kit (Qiagen).

### Mapping of *Snorc* transcriptional start sites

The transcription start sites of the mouse and rat *Snorc* gene were mapped using the oligo capping 5′RACE and 3′RACE strategy as described previously^9,62^. As starting material, PolyA RNA was purified from total RNA extracted from differentiated mouse ATDC5 cells at day 16, and confluent rat chondrosarcoma (RCS) cells were used.

### Cell culture, transient transfection and Luciferase (Luc) assays

All procedures were as previously described in earlier publications from our group^63–65^. The rat chondrosarcoma cells (RCS) were obtained from Dr. Benoit de Crombrugghe (University of Texas, Houston, USA). HEK293 cells were obtained from ATCC and ATDC5 were from Riken. RCS and HEK293 cells were grown in DMEM (Gibco) and supplemented with 10% fetal bovine serum (FBS) (In vitrogen) with 1X PenStrep. ATDC5 were cultured in alpha MEM supplemented with 5% FBS, and differentiated with alpha MEM supplemented with 10% FBS and 50ug/ml ascorbate. For transfection studies cells were seeded in 12-well plate at density of 65000 cells/cm^2^. Twenty-four hours after seeding, medium was changed and cells were transfected with X-tremeGENE 9 (Roche) according to the manufacturer instructions. For single transfections, 400 ng of Luc-reporter plasmid was transfected. For co-transfection studies, a plasmid mix containing 100 ng of Luc-reporter and 300 ng of effector (e.i. transcription factor) were used. Twenty-four hours after transfection, 250μl of passive cell lysis buffer (Promega) was added per well and Luc activity was measured using 5μl of cell extract with 100μl of the luciferase assay system (Promega) on a Sirius luminometer (Berthold, Oak Ridge, TN). Each transfection was done on duplicate wells and repeated at least 4 times. As negative controls, the empty pGL4 basic or a GFP expressing plasmids were used to calculate the induction fold. Mean values with standard errors are either reported as raw RLU measured or by fold induction relative to controls. All experiments were conducted in triplicate wells, and repeated from 4-6 times.

### Immunofluorescence (IF) labeling and western blotting

The procedures for IF detection and western blot analysis of SNORC were essentially as described previously^64,66^. Briefly, cells were fixed with 3% formaldehyde (w/v) in PBS for 10 min at RT°. All following steps were performed for 1 h at RT°. After washing with PBS (3 × 5 min), they washed and processed immediately to IF (non permeabilized) or were permeabilized with Triton X-100 (0.1% in PBS) for 2 min. Cells were blocked with 2% skim milk with 0.1% BSA (w/v) in PBS. The primary (mouse anti-FLAG Origene 1/1000; rabbit anti-SNORC 1/2000) and secondary (anti-rabbit- or anti-mouse-coupled Alexa-Fluor-594 1/1000) antibodies were diluted in the blocking solution. In between incubations antibodies were washed-out with PBS (3 ×5 min). Cells were mounted with Prolong-Gold Antifade reagent with DAPI (Life Technologies). and imaged by epifluorescence on a Leica DMRB microscope equipped with an Olympus DP70 digital camera. For assessment of proteoglycan side chain composition, cells were washed with 100 mM Tris-acetate buffer (pH 7.3) containing 2 mM EDTA and 2U/ml of respective enzymes (chABC, chAC, chB) for 20 min at 37°C and collected (as follows). Otherwise, cells were simply washed with PBS and collected by directly scraping with lysis buffer (50 mM Tris-HCl (pH7.4), 150 mM NaCl, 1 mM EDTA, 1% (v/v) NP40) containing proteases inhibitor cocktail (Sigma). After centrifugation, the soluble fraction was mixed with reducing 4X Laemmli sample buffer, boiled and loaded on SDS-PAGE and processed for western blotting.

### RNA extraction, Northen blotting, reverse transcription (RT), and real-time qPCR (qPCR)

RNA was extracted from tissues or cells with Trizol (Life Technologies). Purified RNA was quantified on a NanoDrop spectrophotometer (Thermo Scientific). Expression was analyzed by Northern blotting or by RT-qPCR essentially as detailed elsewhere^10,66^. For RT-qPCR, the High Capacity cDNA synthesis kit (Applied Biosystems) was used to generate the cDNAs, which were amplified on an Applied Biosystem 7500 PCR machine with the 2X Universal PCR Master Mix and the Taqman probes (Applied Biosystems) for mouse (Mm01287299_m1) and rat (Rn00667869_m1) *Snorc*, and housekeeping beta-actin for mouse (4352933) and rat (Rn00667869_m1). Values are expressed as 2^−ΔCt^.

### Electrophoretic mobility shift assay (EMSA)

Control and hSOX9-containing nuclear extracts used for EMSA were prepared from transiently transfected HEK293 cells based on published methods^63^. For EMSA, oligonucleotide pairs were end labeled with T4 Polynucleotide kinase (NEB) and 50μCi of γ^32^P-ATP (3000 Ci/mmol) (Perkin Elmer) at 37 °C for 1 h. They were then annealed and purified on Illustra ProQuant G50 micro columns (GE Healthcare). Labeled double stranded oligonucleotides (50000 CPM; 13 fmoles) were incubated with nuclear extracts for 20 min at 25 °C and separated on 4% acrylamide-TBE (0.5×) gels. Gels were dried and autoradiography performed for 3 to 15 h.

### Crispr-Cas9-mediated inactivation of the *Snorc* enhancer in RCS cells

Two different gene-editing plasmids were gift from Feng Zhang and obtained from Addgene (#52962 and 52963) (see Fig. 8A). Oligonucleotides for the rat *Snorc* RNA guide (GGCTCTTCATGGCTGTACAAAGG) were synthesized, annealed, and cloned into the BsmBI sites of plasmid 52963. Lentiviruses were produced by transient transfection in HEK293 cells with helper plasmids encoding VSVG and GAG-POL, both gift from Didier Trono (Addgene plasmids #12260 and 12259). The conditioned media containing the viruses were collected after 72-96 h and filtered through 0.45μm. Polybrene was added to final concentration of 8μg/ml and used to infect RCS cells. Stable cells were selected over 2 weeks in the presence of blasticidin (10μg/ml) and puromycin (10μg/ml).

### Chromatin immunoprecipitation (ChIP) assay

The ChIP assay was performed on RCS using the EZ Chip kit (EMD Millipore 17-371) as per vendor instructions. Briefly, confluent RCS cultures were crosslinked for 10 min with freshly prepared formaldehyde (1% final), quenched with 10X glycine, washed with cold PBS, scraped and centrifuged. The cell pellet was resuspended in 2 ml cell lysis buffer and sonicated 7 pulse of 30 sec with 30 sec pause between each (40% amplitude) (Sonic Dismembrator, Model 500, Fisher Scientific). The sheared chromatin was further purified with MinElute reaction cleanup kit (Qiagen), aliquoted and frozen at -80 °C until use. The sheared chromatin was pre-cleared with protein G agarose followed by immunoprecipitation with 6μg of the following antibodies: SOX9 (Millipore AB5535), SOX6 (Millipore Ab5805 and Abcam Ab30455), normal rabbit IgG (SantaCruz sc-2027), or without antibody. Samples were reverse crosslinked, purified on columns, and DNA was eluted with 50μl. Typically, 5μl of the purified material was used in two PCR with primers covering *Snorc* exon 1 or the intronic enhancer region, and the rat aggrecan (*Acan*) A1 enhancer located about 10 kb upstream of exon 1.

### *Snorc*-βGeo construct and cloning

A 434 bp mouse *Snorc* genomic DNA segment was amplified and cloned into pBluescript-KS. It encompassed 187 bp upstream of the coding ATG through to 203 bp downstream of exon 1 within intron 1. The initiator methionine codon (ATG) within exon 1 was next mutated to CCG. The *Snorc* intronic enhancer multimerized 4X copies (4X i68) was inserted (in sense and antisense orientation) at the blunted MscI site located at the 3′ end of the intron 1 segment. The βGeo reporter plasmid^67^ was digested with BglII and KpnI to excise a fragment (4360 bp) covering part of the adenovirus intron with the splice donor, βGeo, and bovine growth hormone polyadenylation site. The latter fragment was subcloned downstream of the *Snorc* intron enhancer. The last cloning gave rise to plasmids with either sense or antisense orientation of the 4X i68 enhancer.

### *Snorc* enhancer βGeo reporter plasmid testing in HEK293 and RCS cells

The βGeo reporter plasmids with and without the 4×75 bp enhancer (sense and antisense) were tested first by transient transfection in HEK293 and RCS cells. HEK293 cells were co-transfected with the Snorc-βGeo and a CMV-driven SOX9 expression plasmid, or the empty control plasmid. After 24 h, the beta-galactosidase activity was measured the Tropix Galacton-Plus chemiluminescent enzyme assay kit (Applied Biosystems). Cellular extracts were prepared with the kit solubilisation buffer and assayed with the Galacton-plus substrate in a Sirius luminometer (Berthold, Oak Ridge, TN). Activity was normalized to total protein content as measured by the Bradford Protein Assay (Bio Rad). For the generation of stably expressing RCS, linear DNA fragments for the *Snorc*-βGeo were excised from the 3 reporter plasmids (no enhancer, 4×75 bp copies sense and antisense) by restriction with XhoI and SpeI. Five μg of DNA was mixed with 0.2 ml cell suspension (2 × 10^6^ RCS cells) and electroporated in a 0.4 mm gap cuvette at 250 V and 500uF at 4 °C using a BioRad GenePulser II unit. Cells were seeded in complete media, and 48 h later, cells were passage and selection was started by addition of G418 (500μg/ml) in the culture media. Selection was maintained for 2 weeks until a stable RCS cell population grew actively. Cells transfected with the reporter lacking the enhancer did not grow.

### *Snorc*-βGeo reporter transgenic mice genotyping and β-galactosidase staining

The *Snorc*-βGeo-sense plasmid was excised of vector sequence by digestion with SpeI and XhoI. The linearized 5192 bp fragment was purified on 1% agarose gel and used for pro-nuclear injections into C57BL6 fertilized egg at the Clinical Research Institute of Montreal (IRCM) transgenic core facility. Transgenic founders were identified by PCR genotyping on gDNA extracted from tail clips. Transgene copy number was assessed by qPCR using Taqman probes for the mouse *Snorc* (Mm01287299_m1) and the endogenous *Tfrc* (4458370) reference. Founders were mated to wild type C57BL6 and embryos were collected at specified developmental stages, day 0.5 denoting the morning of vaginal plug detection and processed for X-gal staining by whole mount or on cryosections as described^65^. Stained cryosections were subsequently counterstained for 10 seconds with 1% eosin Y (w/v in Milli-Q water) then washed in distilled water, dried and mounted. The animal use protocol (AUP5853) and all procedures were reviewed and approved by the Shriners Hospitals for Children Animal Care Committee and the McGill Institutional Animal Care and Use Committee. Shriners Hospitals for Children – Canada and McGill University are accredited by and follow the guidelines of the Canadian Council on Animal Care.

### Immunohistochemistry (IHC) and western blotting for SNORC

Detection of SNORC was performed using a rabbit antibody that was raised against a recombinant bacterial SNORC protein. It consisted of a tandem duplicated (head to tail configuration) extracellular portion of mouse SNORC covering amino acids 25-91 fused to the DHFR-6His coding sequence in the bacterial expression plasmid pQE16 (QIAGEN). The protein was produced in the SG13009[REP4] E. coli strain and purified to homogeneity by nickel-affinity chromatography. The protein was mixed with complete Freund adjuvant and used to immunized 2 rabbits (Pierce Biotechnologies). Total IgG from the exsanguination serum bleeds were affinity purified on protein A-agarose. For IHC, tissue sections were first treated with chondroitinase ABC (2U/ml) for 1 h at 37 °C. Peroxidase activity was quenched using the Dual Endogenous Enzyme Block (DAKO S2003). Sections were blocked with 5% skim milk (w/v) in PBS for 1 h at RT°. The anti-SNORC antibody was applied diluted at 1/5000 in 1% (w/v) skim milk in PBS for 1.5 h at RT°. The anti-rabbit secondary HRP-labeled antibody (EnVision+ HRP-labeled polymer K4002) and revelation substrate (Liquid DAB + Chromogen System K3468) were from DAKO. After staining, sections were slightly counterstained with Hematoxylin QS (Vector Laboratories H3404).

## Supplementary information

Supplementary information.

## Data Availability

All data generated or analyzed during this study are included in this published article and its Supplementary Information file.
