# Supplementary material for: Biochemical characteristics of the chondrocyte-enriched SNORC protein and its transcriptional regulation by SOX9
Source: Sci Rep. 2020 May 8;10:7790. doi: 10.1038/s41598-020-64640-x (PMC7210984; doi:10.1038/s41598-020-64640-x)
Supplement: Supplementary file 1 — Supplementary information. [file 41598_2020_64640_MOESM1_ESM.pdf]

Supplemental data

Biochemical characteristics of the chondrocyte-enriched SNORC protein and its transcriptional regulation by SOX9.

Prashant Kumar Jaiswal[1], Latifa Aljebali[1], Marie-Helene Gaumond[1], Chun-do Oh[4], Hideyo Yasuda[5], Pierre Moffatt[*1,2,3]

[1]Shriners Hospitals for Children – Canada, Montreal, Quebec, Canada

[2]Department of Human Genetics, McGill University, Montreal, Quebec, Canada

[3]Research Institute of the McGill University Health Centre, Montreal, Quebec, Canada.

[4] Department of Orthopedic Surgery, Rush University Medical Center, Chicago, IL 60612

[5] Department of Genetics, University of Texas, MD Anderson Cancer Center, Houston, TX 77030

*To whom correspondence should be addressed:

Shriners Hospitals for Children – Canada

1003 Blvd. Decarie

Montreal, Quebec, Canada H4A 0A9

Tel. 514-282-7161

Email: pmoffatt@shriners.mcgill.ca

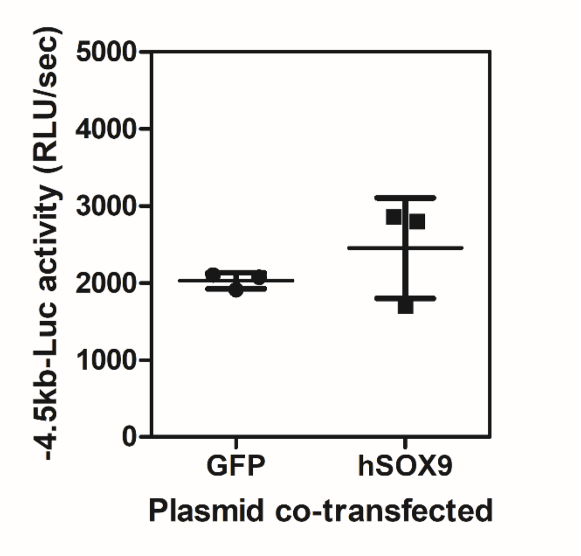

Supplemental Figure 1. The mouse *Snorc* -4.5kb promoter is not induced after co-transfection with hSOX9. HEK293 cells were co-transfected with the mouse -4.5kb-Luc construct in combination with GFP or hSOX9 expression plasmids (100ng each). The luciferase activity was measured 24 hours after transfection. The values represent the raw luciferase activity and each point is the average of triplicate wells from 3 independent experiments (average ± SD). The difference between the 2 conditions (GFP vs hSOX9) is not statistically significant (p=0.16; unpaired Student T-test; one tail).

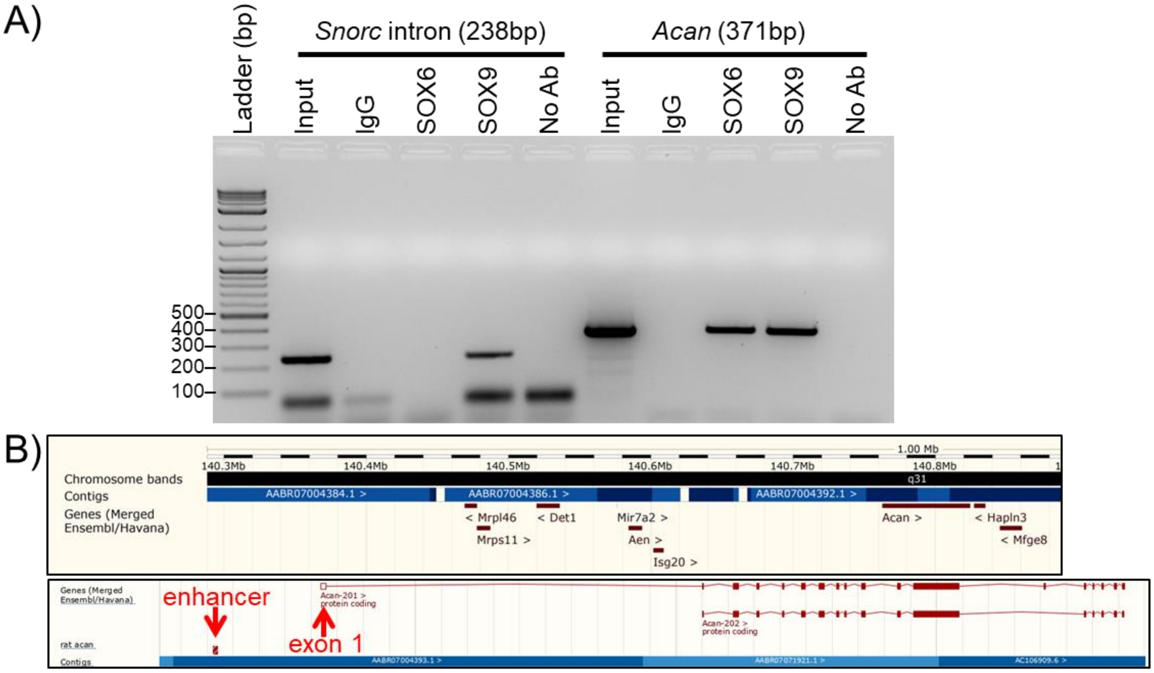

C)

CACAATCCCAGAGAAGTGACCCGGGTCCAGAATGGAAGGGACCAAGGTGCCAGGGATGACCAAGCCCTCAAGAATCCCCCTAGAAGCTCCTCC
CTGGAGACACCCTTCCCCAGAATAGGCAGAGAGGGCTAGAAACACCCATATAAACAGCGCATCCACAAAACCCCACTGTCCCAAGGCTTTCGT
CCCTCAGCCTTTGCTATTTTTATCTGGAGATCGAGAAGGGGGTTGCCAGGCAGGGCCACGCCGCTGTTTATGTCTGAAATTTGAAAAGATGCA
CAGTGTACTTGGGAGAAGGCGTTGCAGGGAAAGGAATTTCAGAGAATTTCAAACCTGGCAGGGTGAATAGGTGCCTTGTGTTCAGGCTTAC

Supplemental Figure 2. Confirmation of the ChIP results obtained from RCS cells. A) Chromatin from RCS was incubated overnight with 8μg of the indicated antibodies: IgG, normal rabbit IgG (SantaCruz sc-2027); SOX6, rabbit anti-SOX6 (Abcam Ab30455); SOX9, rabbit-anti-SOX9 (Millipore AB5535). As a negative control, incubation was also performed without addition of any antibody (No Ab). The positive control is the Input (2% of total). The purified immunoprecipitated chromatin was used for PCR amplification using primers covering the rat *Snorc* intron, and the aggrecan (*Acan*) enhancer. Products were resolved on a 1.8% agarose gel and stained with ethidium bromide (pictured inverted). Representative results of 2 independent experiments. B) Illustration of the rat *Acan* gene (from Ensembl) and its enhancer located about 10kb upstream of exon 1. C) Sequence of the 371bp *Acan* enhancer amplified with locations of primers (underlined), which contains SOX9 and SOX6 binding sites as previously reported by others (Refs. 17, 18).

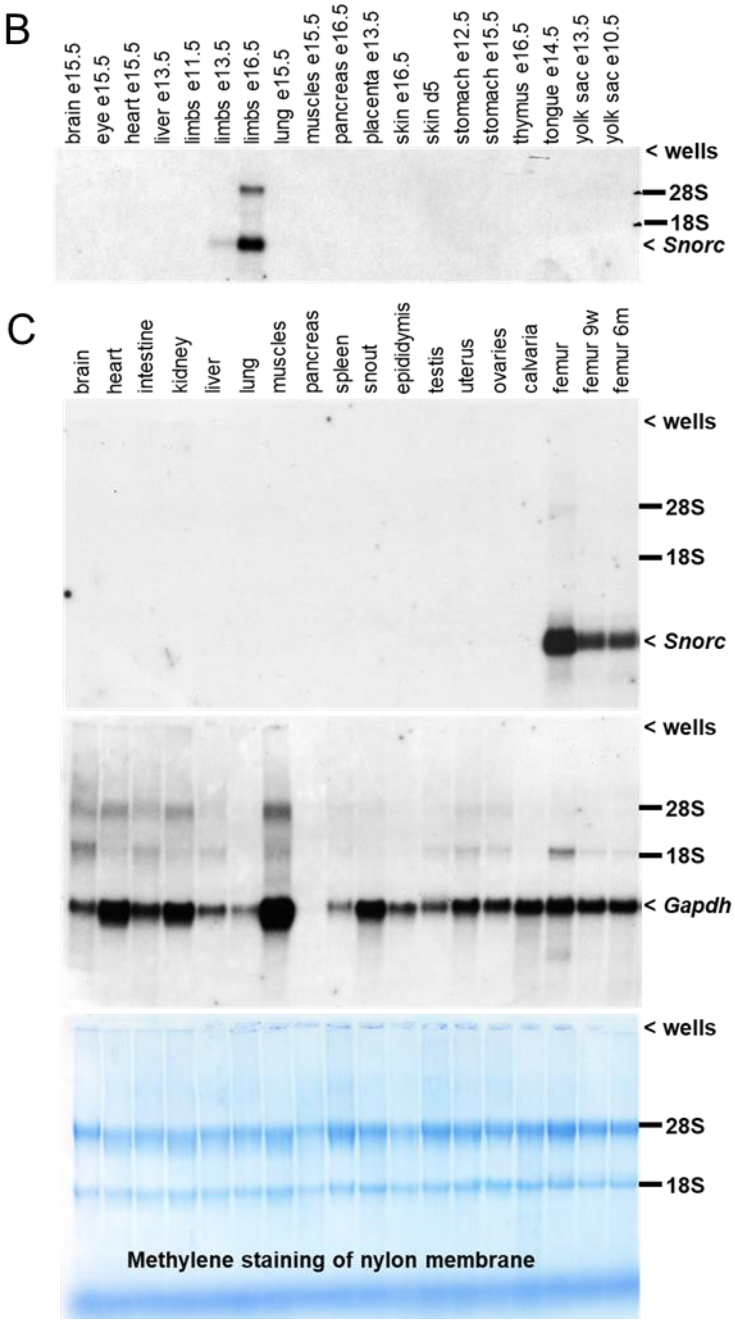

Supplemental to Figure 1. Original autoradiographic films from the Northern blot data as presented in Figure 1 B and C. The position of the 28S and 18S ribosomal RNA and location of loading wells are indicated at right. For panel C), a matching methylene blue staining of the nylon filter is presented at the bottom to show equal loading and relative integrity of ribosomal RNA.

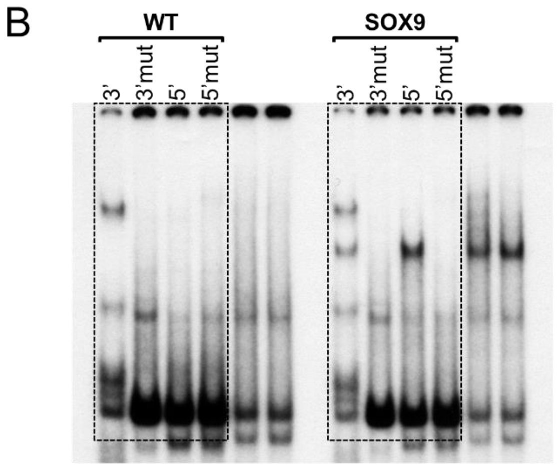

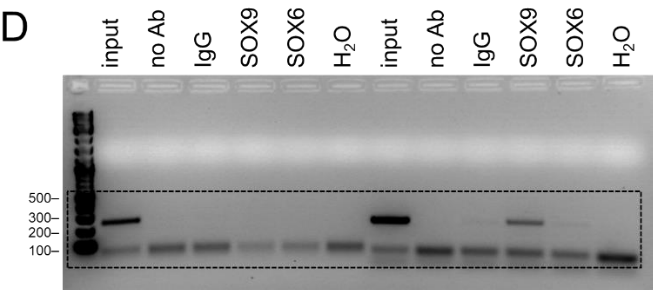

Supplemental to Figure 6. B) Uncropped autoradiographic film used for the EMSA in Figure 6, panel B. D) Uncropped and unprocessed ChIP picture of the ethidium bromide stained agarose gel shown in Figure 6, panel D. Dashed boxes indicate portions used in manuscript Figure 6.

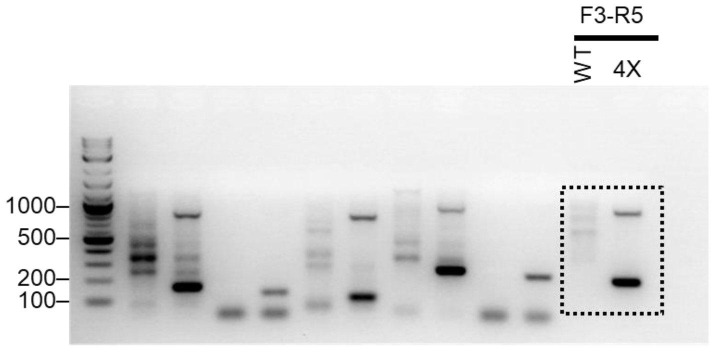

Supplemental to Figure 7. Uncropped and unprocessed image of the ethidium bromide stained agarose gel shown in Figure 7F. Dashed box indicates portion used in manuscript Figure 7.

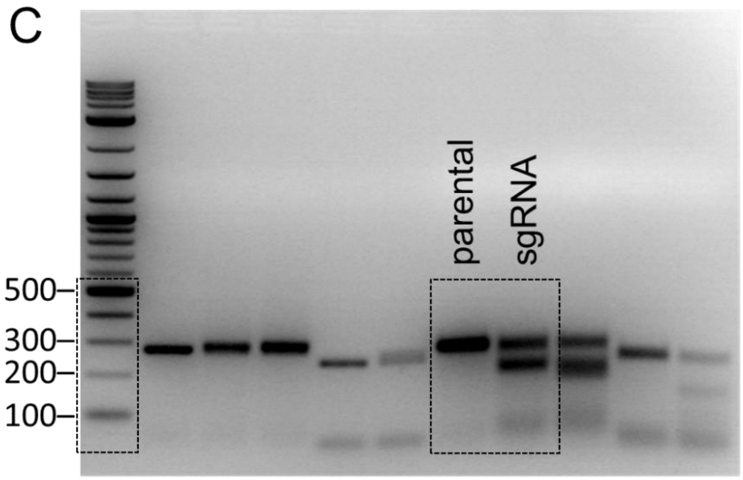

Supplemental to Figure 8. C) Uncropped and unprocessed picture of the ethidium bromide stained agarose gel shown in Figure 8C (surveyor assay). Dashed boxes indicate portions used in manuscript Figure 8.

Supplemental Table 1: List of primers used

Relevance to manuscript	Primer name	Strand	Primer sequence (5'>3')	Specie	Notes
Mouse Snorc cDNA cloning (Figure 1, 2)	mSnorc_F1	FWD	AGACTGCTCACTCCCTGC	mouse	cDNA cloning_5'UTR
	mSnorc_R1	REV	CTTCAAGAAGCGGAAAACTTCC	mouse	cDNA cloning_stop codon underlined
	mSnorc-F-S44A	FWD	CTGGTGAAGGTCCCCTGGA	mouse	mutation of S44A
	mSnorc-R-S44A	REV	CGGGCAATTCAATAGGCTCGT	mouse	mutation of S44A
	mSnorc-DHFR-F	FWD	AGCAGAGGGTCCCCAGGAGC	mouse	bacterial SNORC-DHFR fusion
	mSnorc-DHFR-R	REV	CGCCTGGTCCAAGCGATCCTC	mouse	bacterial SNORC-DHFR fusion
	bact-mSnorc-F-XhoI	FWD	AACTCGAGGCAGAGGGTCCCCAGGAG	mouse	bacterial SNORC_no phusion XhoI site underlined
	mSnorc_Flag_n-term-F	FWD	GGGCCCCAGGAGCCCGATCCCA	mouse	N-term FLAG cloning_ApaI site underlined
	mSnorc_Flag_n-term-R	REV	CTTGTCATCGTCGTCCTTGTAGTCCTCTGCTGTGAGCACCGCAGG	mouse	N-term FLAG underlined
	mSnorc_3XFlag_n-term-F(18-13)	FWD	AACCCACTGCTTACTGGC	mouse	C-term 3XFLAG cloning
	mSnorc_3XFlag_n-term-R(blunt)	REV	GAAGCGGAAAACTTCCTTAGCG	mouse	C-term 3XFLAG cloning
	hNeG186-F1	FWD	CTCACTCCCGGCCAGGAT	human	cDNA cloning
	hNeG186-R1	REV	CGCTTCAGGAGGCAGAAAAC	human	cDNA cloning
Rat Snorc 5'UTR mapping, cDNA cloning, and ORF validation (Figure 4)	rSnorc-5UTR-F5	FWD	AGGACAGAGTTTGCATTGATG	rat	Rat RGD1311447 uATG underlined
	rSnorc-RACE-R3	REV	GCTCTCCAGGGGACCTTCAC	rat	5'RACE-PCR
	rSnorc-RACE-R4	REV	TGGTGCTGGAGCCGAGGTG	rat	5'RACE-RT
	rSnorc-mut-upATG-F	FWD	CAAAACCCTTCCCAGCTCTTTGAA	rat	Rat RGD1311447 uATG mutation
	rSnorc-mut-upATG-R	REV	ATCAATGCAAACTCTGTCCTGG	rat	Rat RGD1311447 uATG mutation
	rSnorc-F1	FWD	CATAACACTCCTCACTCACTGC	rat	rat Snorc
	rSnorc-R1	REV	GGCTTCAAGAAGCAGAAAACTTCC	rat	rat Snorc
Intronic SOX enhancer element mutation and deletion (Figure 5)	m_promo_-4548bp_FWD	FWD	AATGTCCCTCGTCTTGGTGA	mouse	4548bp mouse promoter
	m_promo_-4548bp_REV	REV	CCTGGCAGGGAGTGAGCA	mouse	4548bp mouse promoter
	68_promo-F4	FWD	CTTTCCCGCTGCCTGAGCCCAG	mouse	Snorc 5'UTR -68
	68_promo-R5	REV	CTAGCGAGCTCAGGTACCGGC	mouse	Snorc 5'UTR -68_within pGL4-basic
	mSnorc_i847_F6	FWD	AGCAGCAGGATGGGTGGC	mouse	i847 intronic fragment
	mSnorc_i847_R6	REV	CCCAAAATCCATTCGGGCTT	mouse	i847 intronic fragment
	mSnorc_i758_R2	REV	ATAGGCCAGTGTGCCAGGAGGAG	mouse	i758 intronic fragment
	mSnorc_i758_F2	FWD	ATAGCCAAGACCTCGCCTGACC	mouse	i758 intronic fragment
	mSnorc_i63_F	FWD	AGTCTAGAGGCCAGGGTGGGGATGAAAAG	mouse	i63 SOX9 enhancer_XbaI underlined
	mSnorc_i63_R	REV	GAGCTAGCCCATGCTCACAGACGGCCT	mouse	i63 SOX9 enhancer_NheI underlined
	mSnorc_pointmut5'-F	FWD	CGAGGCTCTTCATGTCTGCACAAAGG	mouse	point mutation 5'SRY i758 intronic fragment
	mSnorc_pointmut5'-R	REV	GCATCCCCACCCTGGCCTCCTGC	mouse	point mutation 5'SRY i758 intronic fragment
	mSnorc_pointmut3'-F	FWD	GCGGCCGTCTGTGAGCATGGGC	mouse	point mutation 3'SRY i758 intronic fragment
	mSnorc_pointmut3'-R	REV	CGTGCAGACATGAAGAGCCTTTTCATC	mouse	point mutation 3'SRY i758 intronic fragment
	mSnorc_delta3'-F	FWD	AGCATGGGCGCCTGGCCTGG	mouse	deletion 3'SRY i758 intronic fragment
	mSnorc_delta3'-R	REV	CAGACATGAAGAGCCTTTTCATCC	mouse	deletion 3'SRY i758 intronic fragment
	mSnorc_delta5'-F	FWD	GTCTGCACAAAGGCCGTCTGTGAG	mouse	deletion 5'SRY i758 intronic fragment
	mSnorc_delta5'-R	REV	ATCCCCACCCTGGCCTGGCTG	mouse	deletion 5'SRY i758 intronic fragment
	hSNORC_i427_F	FWD	AGCTCTGGACAGGTGTGTAC	human	i427 intronic enhancer
	hSNORC_i427_R	REV	CCCAGACTCCACTGACCAG	human	i427 intronic enhancer
	hSNORC_i427_delta-SRY_F	FWD	AGCAGAGGCGCCTGGCCTC	human	i427 intronic enhancer
	hSNORC_i427_delta-SRY_R	REV	ATCCCTGCCCTGGCCTCCT	human	i427 intronic enhancer
Emsa and ChIP (Figure 6)	mSnorc_SRY5'_GS_F	FWD	GGGATGAAAAGGCTCTTCATGTCTGCA	mouse	EMSA
	mSnorc_SRY5'_GS_R	REV	TGCAGACATGAAGAGCCTTTTCATCCC	mouse	EMSA
	mSnorc_SRY3'_GS_F	FWD	TCTGCACAAAGGCCGTCTGTGAGCATG	mouse	EMSA
	mSnorc_SRY3'_GS_R	REV	CATGCTCACAGACGGCCTTTGTGCAGA	mouse	EMSA
	mSnorc_SRY-5'mut-GS-F	FWD	GGGATGCCGAGGCTCTTCATGTCTGCA	mouse	EMSA mutation underlined
	mSnorc_SRY-5'mut-GS-R	REV	TGCAGACATGAAGAGCCTCGGCATCCC	mouse	EMSA mutation underlined
	mSnorc_SRY-3'mut-GS-F	FWD	TCTGCACGCGGGCCGTCTGTGAGCATG	mouse	EMSA mutation underlined
	mSnorc_SRY-3'mut-GS-R	REV	CATGCTCACAGACGGCCCGCGTGCAGA	mouse	EMSA mutation underlined
	rSnorc_5'UTR_F3	FWD	CCAGGAGGACAGAGTTTGCA	rat	ChIP exon 1
	rSnorc_intron_R10	REV	TGGTAATTGTTGGCCCTGAG	rat	ChIP exon 1
	rSnorc_intron_R9	REV	GCTCAACGGTTGGTCTGACCAC	rat	ChIP intron 1
	rSnorc_intron_F11	FWD	TGGGCTGCCGTCTTGTAGCAG	rat	ChIP intron 1
bGeo reporter (Figures 7 and 9)	mSnorc_exon1_F3	FWD	TGGCCCCTGCGGTGCTCAC	mouse	RT-PCR
	bGeo_LacZR5	REV	GCCTCTTCGCTATTACGCC	bGeo	RT-PCR
	mSnorc_mutATG-F	FWD	CGGCATCTTGTCTGGCCCTGCGC	mouse	mutation of ATG>CCG
	mSnorc_mutATG-R	REV	GCCTGGCAGGGAGTGAGCAGTCT	mouse	mutation of ATG>CCG
	mSnorc_geno_F1	FWD	TGCGCGTGCATGTGTGAG	mouse	genomic segment for transgenic
	mSnorc_intron_R1	REV	TGGCCACCCATCCTGCTGCTAAC	mouse	genomic segment for transgenic
	bGeo_LacZR4	REV	ACGACGGGATCCGCCATGTC	bGeo	genotyping transgenic lines
	mSnorc_intron_F2	FWD	ATAGCCAAGACCTCGCCTGACC	mouse	genotyping transgenic lines
Crispr guide RNA to target rat Smpec enhancer (Figure 8)	rSnorc_sgRNA_i1_#5_F	FWD	CACCGGCTCTTCATGGCTGTACAA	rat	underlined BsmBI overhang
	rSnorc_sgRNA_i1_#5_R	REV	AAACTTGTACAGCCATGAAGAGCC	rat	underlined BsmBI overhang
	rSnorc_intron_R9	REV	GCTCAACGGTTGGTCTGACCAC	rat	amplification for sequencing and Surveyor assay
	rSnorc_intron_F10	FWD	TGCGGGTGTGTCTAAGGTCTG	rat	amplification for sequencing and Surveyor assay

Supplemental Table 2: List of plasmids used

Plasmid name	Source	Specie	Notes/reference	Notes
hSOX9	Veronique Lefevbre	human	Lefebvre et al. (1997) Mol Cell Biol 17:2336-2346.	expresses human FLAG-tagged-SOX9 protein under CMV promoter and bGH pA
mSOX6	Veronique Lefevbre	mouse	Lefebvre et al. (1998) EMBO J. 17(19):5718-33	expresses mouse FLAG-tagged-SOX6 protein under CMV promoter and bGH pA
mSNORC	current manuscript	mouse	CMV-based	cloned from mouse E16.5 limb
rSNORC	current manuscript	rat	CMV-based	cloned from rat RCS cells
hSNORC	current manuscript	human	CMV-based	cloned from human U2OS
lentiCas9-Blast	Addgene 52962	n/a	gift from Feng Zhang	Expresses human codon-optimized S. pyogenes Cas9 protein and blasticidin resistance from EFS promoter. 3rd generation lentiviral backbone.
lentiGuide-Puro	Addgene 52963	n/a	gift from Feng Zhang	Expresses S. pyogenes CRISPR chimeric RNA element with customizable sgRNA from U6 promoter and puromycin resistance from EF-1a promoter. 3rd generation lentiviral backbone
SA-betaGeo	Addgene 21709	n/a	Philippe Soriano/ Genes Dev. 1991 Sep . 5(9):1513-23.	bifunctional lacZ/neomycin phosphotransferase gene
pQE-16	QIAGEN (cat # 32903)	n/a		DHFR-6His
GD261_3X FLAG tag		n/a		CMV-based cloning plasmid
GD162-GFP	pQBIfc3 (Qbiogene)	n/a		GFP expressing plasmid (used as negative controls in co-transfection experiments)'
pCMV_3.1	Invitrogen	n/a		empty control plasmid - CMV-base (bGH_pA)
pGL4.10[luc2]	Promega E6651		luciferase reporter	The pGL4.10[luc2] Vector encodes the luciferase reporter gene luc2 (Photinus pyralis) and is designed for high expression and reduced anomalous transcription. The pGL4 Vectors are engineered with fewer consensus regulatory sequences and a synthetic gene, which has been codon optimized for mammalian expression. The pGL4.10[luc2] Vector is a basic vector with no promoter. However, it contains a multiple cloning region to allow cloning of a promoter of choice.
minus89col2	Veronique Lefevbre	mouse	Lefebvre et al. (1996) Mol Cell Biol. 16(8):4512-23	pGL2-based plasmid - mouse col2a1 promoter -89bp
minus89col2-4x48bp enhancer	Veronique Lefevbre	mouse	Lefebvre et al. (1996) Mol Cell Biol. 16(8):4512-23	pGL2-based plasmid - mouse col1a2 promoter -89bp with 4 copies of the 48bp col2a1 enhancer
psPax2	Addgene 12260	n/a	gift from Didier Trono	2nd generation lentiviral packaging plasmid. Can be used with 2nd or 3rd generation lentiviral vectors and envelope expressing plasmid (Addgene#12259)
pMD2.G	Addgene 12259	n/a	gift from Didier Trono	VSVG Envelop plasmid
